# No sex difference was found in the safety and efficacy of intravenous alteplase before endovascular therapy

**DOI:** 10.3389/fneur.2022.989166

**Published:** 2022-11-09

**Authors:** Min Fang, Chenhaoyi Xu, Lan Ma, Yue Sun, Xiaoyu Zhou, Jiangshan Deng, Xueyuan Liu

**Affiliations:** ^1^Department of Neurology, Shanghai Tenth People's Hospital of Tongji University, Shanghai, China; ^2^Department of Radiology, Chinese Academy of Medical Sciences & Peking Union Medical College, Beijing, China; ^3^Department of Neurosurgery, Shanghai Jiao Tong University Affiliated Sixth People's Hospital, Shanghai, China

**Keywords:** acute ischemic stroke, endovascular therapy (EVT), functional status, intravenous alteplase, sex characteristics

## Abstract

**Background and purpose:**

Prior studies on sex disparities were *post-hoc* analyses, had limited treatment modalities, and had controversial findings. Our study aimed to examine whether sex difference modifies the effect of intravenous alteplase before endovascular therapy.

**Methods:**

We conducted a multicenter prospective cohort study of 850 eligible patients with acute ischemic stroke who underwent endovascular therapy. A propensity score was utilized as a covariate to achieve approximate randomization of alteplase pretreatment. The baseline characteristics of women and men were compared. Logistic regression with interaction terms, adjusted for potential confounders, was used to investigate the effect of sex on the prognosis of bridging therapy.

**Results:**

In comparison to men, women were older [78.00 (70.00–84.00) vs. 67 (61.00–74.00), *P* < 0.001], had more atrial fibrillation (61.4 vs. 35.2%, *P* < 0.001), had a lower ASPECTS [10.00 (8.00–10.00) vs. 10 (9.00–10.00), *P* = 0.0047], and had a higher NIHSS score [17.00 (14.00–20.00) vs. 16 (13.00–19.00), *P* = 0.005]. Women tended to receive less bridging therapy (26.3 vs. 33%, *P* = 0.043) and more retrieval attempts [2.00 (1.00–2.00) vs. 1 (1.00–2.00), *P* = 0.026]. There was no sex difference in functional independence at 90 days after bridging therapy (OR 0.968, 95% CI 0.575–1.63), whereas men benefited more after EVT alone (OR 0.654, 95% CI 0.456–0.937). There were no sex-treatment interactions observed regardless of the location of the occlusion. There were no significant sex differences in all safety outcomes.

**Conclusion:**

Our study could not confirm that sex modifies the treatment effect of intravenous alteplase before endovascular therapy. At the same time, we advocate for women to seek timely medical treatment.

## Introduction

The safety and efficacy of intravenous alteplase before endovascular therapy (EVT) for patients with acute ischemic stroke have been debated (1–3). Previous studies demonstrated that bridging therapy (intravenous alteplase before EVT) was beneficial for patients undergoing endovascular therapy for a large vessel occlusion in the anterior circulation (4, 5). However, intravenous alteplase before EVT may delay the time to initiate EVT and increase the risk of hemorrhagic complications. The DEVT randomized clinical trial (RCT) (6) and the DIRECT-MT clinical trial (7) showed that endovascular thrombectomy alone was non-inferior to bridging therapy, while the SKIP randomized clinical trial (8) and MR CLEAN-NO IV (9) did not. A meta-analysis of three randomized controlled trials found no differences in functional independence of IV thrombolysis-eligible patients with an acute large vascular occlusion undergoing direct EVT compared to bridging therapy (10). Meanwhile, a series of studies attempted to identify differences in outcomes after bridging therapy among different subgroups of patients based on their baseline characteristics, including the location of the occlusion, the volume of the ischemic score, and the National Institutes of Health Stroke Scale (NIHSS) score on admission (11–14). However, studies on the prognostic impact of patient background factors on patients after bridging treatment remain scarce.

Sex is an unchangeable risk factor for stroke. Sex differences in the incidence and development of stroke have been confirmed by research. Women have a higher incidence of stroke because of a longer life expectancy and an older age at the onset of stroke (15). Various pregnancy complications and oophorectomy increase the risk of stroke in women (16). The more severe the degree of stroke, the higher the incidence of atrial fibrillation, and more pre-stroke functional limitations lead to a higher rate of mortality in women (17). Sex differences in clinical outcomes in patients with acute ischemic stroke after endovascular treatment (EVT) have also been discussed in a great number of studies. Some of them suggested that women suffered from poor functional outcomes and were more likely to die, whereas others came up with the opposite conclusion or considered sex to be non-influential on clinical outcomes after EVT (18–22). The explanation for studies with contradictory results might be selection, evident in different baseline characteristics (23, 24). In addition, different confounding variables were included, resulting in inadequate corrections.

Whether sex difference affects the safety and efficacy of intravenous alteplase before EVT has not been discussed. In this prospective cohort study, we studied the effect of sex differences on the prognosis of EVT alone and bridging therapy separately. In addition, the interaction between sex and these treatment modalities was analyzed. Based on the location of the occlusion in the anterior and posterior circulation, a subgroup analysis was performed. We hypothesize that sex might not affect the prognosis of adjunctive alteplase therapy.

## Methods

### Standard protocol approvals, registrations, and patient consent

We conducted a prospective cohort study of patients with AIS who underwent EVT at 3 comprehensive stroke centers in China between January 2017 and September 2019. The study was registered and approved by the local institutional review board. Written informed consent was obtained from all patients.

### Sample size analysis

A power analysis was performed. Prior frequencies of sex and clinical characteristics were estimated from 95 patients enrolled in Shanghai Tenth People's Hospital between 2017 and 2018. The threshold for a significant level was set at 0.05. Sample size and corresponding power were estimated using the chi-square test function in a pwr R-package. The study had more than 75% power for the primary outcome (Supplementary Figure S1).

### Patient selection

The patients' inclusion criteria were (1) adult patients (age ≥ 18 years old) diagnosed with AIS, (2) time to hospital from clinical onset within 24 h, (3) NIHSS score on admission ≥6, (4) occlusion in intracranial arteries including the anterior and posterior circulation according to digital subtraction angiography (DSA), (5) Alberta stroke program early CT score (ASPECTS) or posterior circulation Alberta stroke program early CT score (pc-ASPECTS) on admission ≥6, and (6) clinical outcome follow-up reports available at 3 months. Patients were excluded from the study if they were pregnant or had a myocardial infarction (within 1 month before the study), severe liver or kidney disease, malignant tumors, and blood diseases.

### Variables of interest and outcomes

We recorded the demographic and clinical characteristics of patients at the time of admission. Baseline characteristics included age; sex; blood pressure on admission; smoking and drinking habits; history of taking statins, anticoagulants, and antiplatelet drugs; CHA2DS2-VASc score, modified Rankin scale (mRS) score, NIHSS score, and ASPECTS or pc-ASPECTS on admission; the location of the occlusion; and the TOAST type of stroke. We included coronary heart disease, hypertension, diabetes, atrial fibrillation, and a history of ischemic stroke as comorbidities. Key factors during the treatment process, such as pretreatment with IVT, the number of retrieval attempts, and the EVT modality, were also considered. The time from onset to perform CT (computed tomography) examination, onset to groin puncture, groin puncture to recanalization, onset to recanalization, and performing CT examinations to recanalization were recorded. Furthermore, laboratory data, including platelet count, volume on admission, and neutrophil-lymphocyte ratio (NLR) on admission, were collected.

The primary outcome was functional independence defined as mRS ≤2 at 3 months after EVT. The secondary outcomes included successful reperfusion, defined as final modified thrombolysis in cerebral infarction (mTICI) of 2b to 3, and early neurological improvement, defined as a ≥4 point decrease in NIHSS score 24 h after EVT compared with the NIHSS score on admission. Safety outcomes were intracranial hemorrhage and symptomatic intracerebral hemorrhage (sICH) during the hospitalization and death within 90 days after the treatment. Symptomatic intracerebral hemorrhage was defined as new intracranial hemorrhage detected by brain imaging and associated with ≥4 points in total NIHSS at the time of diagnosis compared to immediately before worsening or ≥2 points in one NIHSS category or the need for major medical/surgical intervention or the absence of an alternative explanation for deterioration (25).

### Statistical analysis

In terms of statistical description, categorical variables were presented as numbers and percentages, whereas continuous variables were expressed as either mean and SD or median and interquartile range.

The missing values of baseline variables were attributed to multiple imputations. Baseline data were presented according to sex (Table 1). The probability of each patient undergoing bridging therapy (the propensity score), which was utilized to achieve approximate randomization of alteplase pretreatment, was obtained from a logistic regression model. Selected covariates were age; sex; blood pressure on admission; smoking and drinking habits; history of taking statins, anticoagulants, and antiplatelet drugs; the CHA2DS2-VASc score; modified Rankin scale (mRS) score; the NIHSS score; ASPECTS or pc-ASPECTS on admission; the history of coronary heart disease, hypertension, diabetes, atrial fibrillation, and ischemic stroke; the location of the occlusion; the TOAST type of stroke; time from onset to perform CT (computed tomography) examination; platelet count; platelet volume; and NLR on admission (Supplementary Table S1). To assess the association of sex and primary and secondary outcomes, logistic regression models, which were adjusted for age, diabetes, history of atrial fibrillation and taking antiplatelet drugs, smoking and drinking habits, CHA2DS2-VASc score, NIHSS score, and ASPECTS or pc-ASPECTS on admission, the location of the occlusion, the TOAST type of stroke, the endovascular treatment modality, and the retrieval attempts during EVT, were used. The calculated propensity scores were included in the adjusted models to achieve approximate randomization of alteplase pretreatment. We also investigated the interaction of sex and treatments by adding multiplicative interaction terms into adjusted logistic regression models. For safety outcomes, the chi-square test or the Fisher exact test was used as appropriate. For the report, ORs with 95% CIs were used, and a *p*-value of <0.05 was considered statistically significant. For subgroup analysis, we divided the patients into two groups based on the location of the occlusion. All analyses were performed using the R statistics program (version 4.1.1, R Core Team 2021, Vienna, Austria).

**Table 1:** Baseline characteristics of male and female patients.

**Baseline characteristics**	**Male**	**Female**	***p***
	***n* = 500**	***n* = 350**	
Bridging Therapy (%)	165 (33.0)	92 (26.3)	0.043
Age, years, [median (IQR)]	67.00 [61.00, 74.00]	78.00 [70.00, 84.00]	<0.001
Hypertension (%)	337 (67.4)	241 (68.9)	0.709
Diabetes (%)	138 (27.6)	75 (21.4)	0.05
Atrial fibrillation (%)	176 (35.2)	215 (61.4)	<0.001
History of stroke or TIA (%)	97 (19.4)	63 (18.0)	0.671
History of coronary artery disease (%)	78 (15.6)	52 (14.9)	0.842
Current smoking (%)	99 (19.8)	2 (0.6)	<0.001
Current drinking (%)	48 (9.6)	1 (0.3)	<0.001
Previous use of antiplatelet (%)	39 (7.8)	44 (12.6)	0.029
Previous use of anticoagulant (%)	22 (4.4)	27 (7.7)	0.06
Previous use of statins (%)	18 (3.6)	21 (6.0)	0.141
CHA2DS2-VASc score on admission [median (IQR)]	2.00 [1.00, 3.00]	4.00 [2.00, 5.00]	<0.001
mRS on admission [median (IQR)]	5.00 [5.00, 5.00]	5.00 [5.00, 5.00]	0.259
ASPECT or pc-ASPECT on admission [median (IQR)]	10.00 [9.00, 10.00]	10.00 [8.00, 10.00]	0.047
NIHSS score on admission [median (IQR)]	16.00 [13.00, 19.00]	17.00 [14.00, 20.00]	0.005
Systolic blood pressure on admission mmHg [median (IQR)]	143.00 [131.00, 159.00]	144.00 [131.00, 159.00]	0.619
Diastolic blood pressure on admission mmHg [median (IQR)]	85.00 [76.00, 90.00]	82.00 [73.00, 90.00]	0.077
Platelet number on admission [median (IQR)]	176.00 [149.00, 219.25]	185.50 [150.25, 223.00]	0.485
Platelet volume on admission [median (IQR)]	10.70 [10.00, 11.40]	10.70 [10.10, 11.50]	0.289
Neutrophil to lymphocyte ratio (NLR) on admission [median (IQR)]	7.80 [4.12, 13.60]	7.60 [4.03, 13.40]	0.529
Anterior circulation occlusion (%)	0.83 (0.38)	0.92 (0.27)	<0.001
Toast classification (%)			<0.001
Large-artery atherosclerosis	252 (50.4)	106 (30.3)	
Cardioembolism	190 (38.0)	223 (63.7)	
Stroke of other determined etiology	20 (4.0)	1 (0.3)	
Stroke of undetermined etiology	38 (7.6)	20 (5.7)	
Time from onset to perform CT, min, (median [IQR])	110.00 [72.00, 184.00]	111.00 [70.00, 180.00]	0.969
Time from onset to groin puncture, min, (median [IQR])	234.50 [180.00, 300.00]	235.00 [163.25, 300.00]	0.457
Time from groin puncture to recanalization, min, (median [IQR])	58.50 [40.00, 78.25]	58.50 [40.00, 80.00]	0.512
Time from performing CT to recanalization, min, (median [IQR])	90.00 [58.50, 131.50]	91.00 [60.00, 127.75]	0.985
Time from onset to recanalization, min, (median [IQR])	302.00 [230.00, 373.25]	301.50 [220.00, 372.00]	0.65
Endovascular treatment modality (%)			<0.001
Arterial thrombolysis	8 (1.6)	3 (0.9)	
Direct mechanical thrombectomy	349 (69.9)	298 (85.1)	
Angioplasty and stenting	106 (21.2)	33 (9.4)	
Angioplasty without thrombectomy	36 (7.2)	16 (4.6)	
Retrieval attempts (median [IQR])	1.00 [1.00, 2.00]	2.00 [1.00, 2.00]	0.026

## Results

### Baseline characteristics

**Figure 1:**
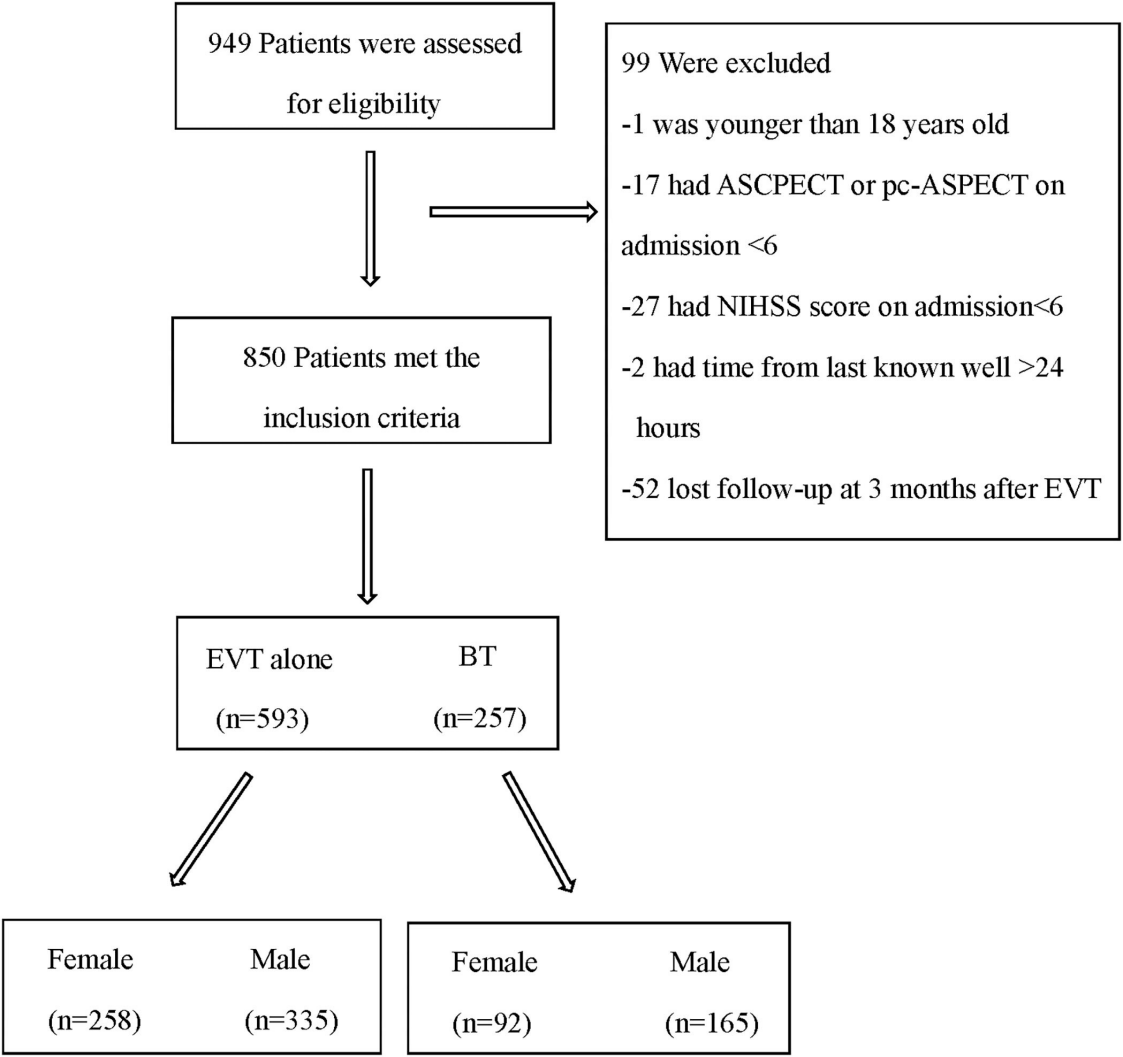
Flowchart of patients in this study. EVT, endovascular therapy; BT, bridging therapy; ASPECT, Alberta stroke program early CT score (ASPECTS); pc-ASPECTS, posterior circulation Alberta stroke program early CT score; NIHSS, National Institutes of Health Stroke Scale.

During the study period, 949 patients from three centers were screened for eligibility, and 850 patients were selected (Figure 1). Baseline characteristics are presented in Table 1. According to our statistics, women were less likely to receive bridging therapy (26.3 vs. 33%, *p* = 0.043). Women were at a higher risk of stroke according to the CHA2DS2-VASc score on admission [4.00 (2.00–5.00) vs. 2 (1.00–3.00), *P* < 0.001] as they were older [78.00 (70.00–84.00) vs. 67 (61.00–74.00), *P* < 0.001] and had more atrial fibrillation (61.4 vs. 35.2%, *P* < 0.001). In addition, women had a lower ASPECTS on admission [10.00 (8.00–10.00) vs. 10 (9.00–10.00), *P* = 0.0047], had a higher NIHSS score [17.00 (14.00–20.00) vs. 16 (13.00–19.00), *P* = 0.005] on admission, and received more retrieval attempts [2.00 (1.00–2.00) vs. 1 (1.00–2.00), *P* = 0.026] than men.

### Effect of sex difference on the primary outcomes

**Figure 2:**
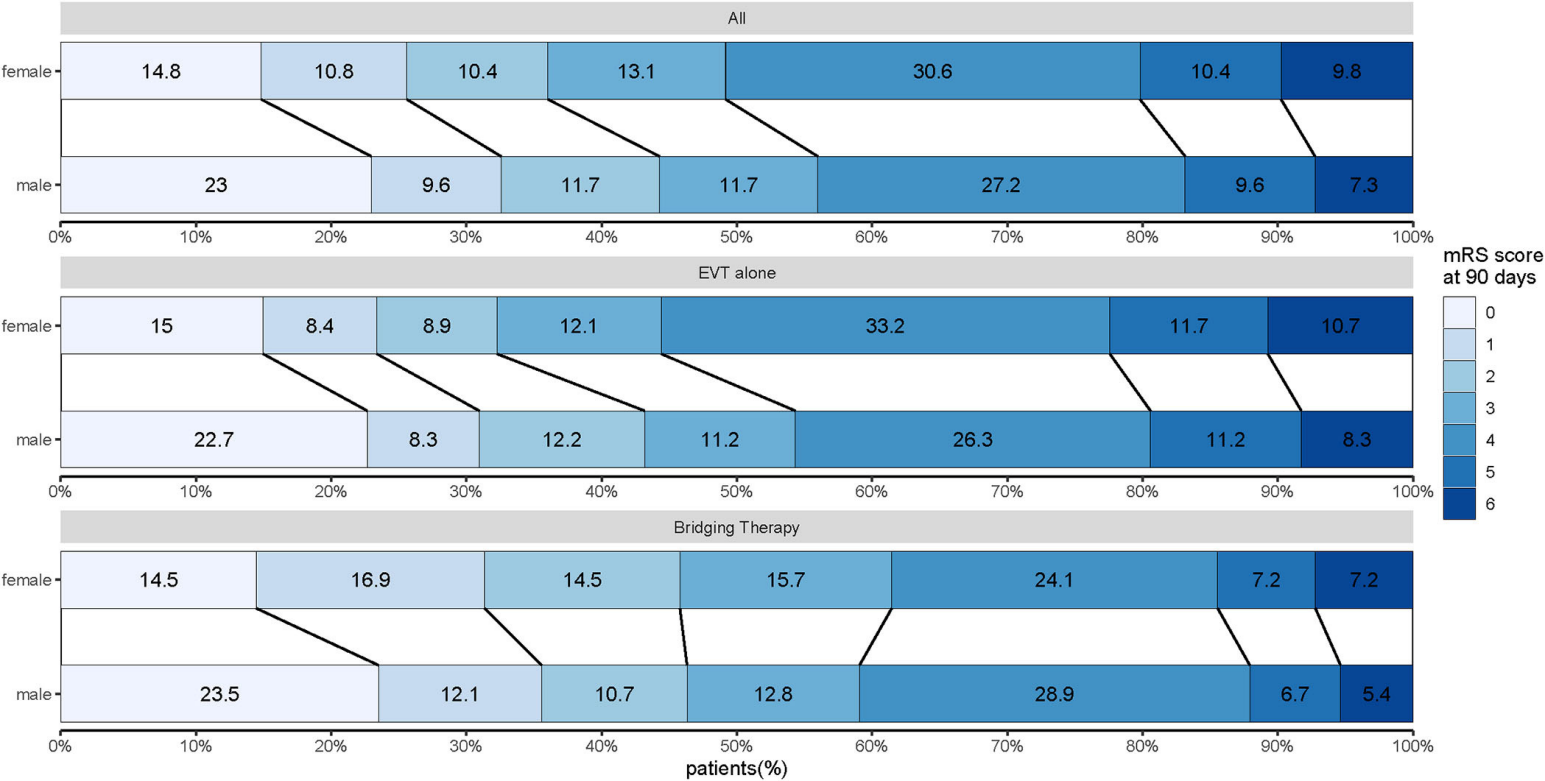
Distribution of modified Rankin scale scores at 90 days for women and men treated with EVT alone and bridging therapy. Scores range from 0 to 6, with 0 indicating no symptoms, 1 indicating no clinically significant disability, 2 indicating slight disability (patient can function without assistance but cannot carry out all previous activities), 3 indicating moderate disability (patient requires some help but can walk unassisted), 4 indicating moderately severe disability (patient cannot attend to bodily needs without assistance and cannot walk unassisted), 5 indicating severe disability (patient requires constant nursing care and attention), and 6 indicating death.

In patients treated with bridging therapy, the functional status of 90 days was not impacted by sex (OR 0.968, 95% CI 0.575–1.63). However, in patients treated with EVT alone, there was a significant association between sex and functional independence at 90 days (OR 0.654, 95% CI 0.456–0.937). While there was insufficient statistical evidence to support an interaction between sex and these two treatments (P _interaction_ = 0.226) (Figure 2; Table 2), we found differences in the effect of sex on EVT alone and bridging therapy for the primary outcomes.

**Table 2:** Sex differences in primary and secondary outcomes after being adjusted.

**Outcomes**	**Treatment**	**Female**	**Male**	**Adjusted effect OR (95%CI)**	***P*-value**	**P (interaction)**
**Primary**						
mRS 0–2 90 days after EVT, *n* (%)						0.226
	EVT alone	68 (26.4)	120 (36.4)	0.654 (0.456–0.937)	0.021	
	Bridging therapy	48 (31.8)	69 (42.6)	0.968 (0.575–1.63)	0.902	
**Secondary**						
Final mTICI 2b to 3, *n* (%)						0.357
	EVT alone	216 (83.7)	289 (87.6)	0.723 (0.453–1.153)	0.173	
	Bridging therapy	79 (86.8)	139 (85.6)	1.087 (0.512–2.307)	0.828	
4 points decrease in NHISS score 24 h after EVT, *n* (%)						0.733
	EVT alone	158 (61.2)	195 (59.1)	1.119 (0.801–1.565)	0.51	
	Bridging therapy	65 (71.4)	108 (66.7)	1.249 (0.714–2.188)	0.436	

mTICI, modified thrombolysis in cerebral infarction.

### Effect of sex difference on the secondary outcomes

In patients treated with EVT alone or bridging therapy, the success rate of reperfusion was similar in both men and women (EVT alone: OR 0.723, 95% CI 0.453–1.153; bridging therapy: OR 1.087, 95% CI 0.512–2.307). There was no significant interaction between sex and treatments (P _interaction_ = 0.357). Sex also had no effect on early neurological improvement (EVT alone: OR 1.119, 95% CI 0.801–1.565; bridging therapy: OR 1.249, 95% CI 0.714–2.188), and there was no interaction about treatment based on sex (P _interaction_ = 0.733) (Table 2).

### Effect of sex difference on the safety outcomes

Women had a similar incidence of intracranial hemorrhage during hospitalization after bridging therapy compared with men (52.7 vs. 40.7%, *p* = 0.066). This result remained constant when women were treated with EVT alone (45.3 vs. 42.4%, *p* = 0.478). The difference in terms of SICH was not significant for women and men (EVT alone: 8.9 vs. 7.9%, *p* = 0.652; bridging therapy: 4.4 vs. 6.8%, *p* = 0.442). Similar deaths were observed within 90 days after treatment (EVT alone: 21.3 vs. 20.6%, *p* = 0.833; bridging therapy: 15.4 vs. 11.7%, *p* = 0.409) (Table 3).

**Table 3:** Safety events in women and men.

**ALL patients**	**EVT alone**			**Bridging therapy**		
**Safety outcomes**	**Female**	**Male**	***P*-value**	**Female**	**Male**	***P*-value**
Intracranial hemorrhage, *n* (%)	117 (45.3)	140 (42.4)	0.478	48 (52.7)	66 (40.7)	0.066
Symptomatic intracerebral hemorrhage, *n* (%)	23 (8.9)	26 (7.9)	0.652	4 (4.4)	11 (6.8)	0.442
Deaths within 90 days after EVT, *n* (%)	55 (21.3)	68 (20.6)	0.833	14 (15.4)	19 (11.7)	0.409
**Anterior circulation**						
Intracranial hemorrhage, *n* (%)	107 (46.3)	113 (43.6)	0.55	47 (53.4)	60 (43.5)	0.146
Symptomatic intracerebral hemorrhage, *n* (%)	20 (8.7)	20 (7.7)	0.706	4 (4.5)	9 (6.5)	0.536
Deaths within 90 days after EVT, *n* (%)	46 (19.9)	50 (19.3)	0.866	13 (14.8)	13 (9.4)	0.222
**Posterior circulation**						
Intracranial hemorrhage, *n* (%)	9 (39.1)	21 (34.4)	0.688	1 (33.3)	5 (21.7)	0.657
Symptomatic intracerebral hemorrhage, *n* (%)	3 (13)	5 (8.2)	0.503	0 (0)	1 (4.3)	0.998
Deaths within 90 days after EVT, *n* (%)	9 (39.1)	17 (27.9)	0.322	1 (33.3)	6 (26.1)	0.791

### Subgroup analysis

**Figure 3:**
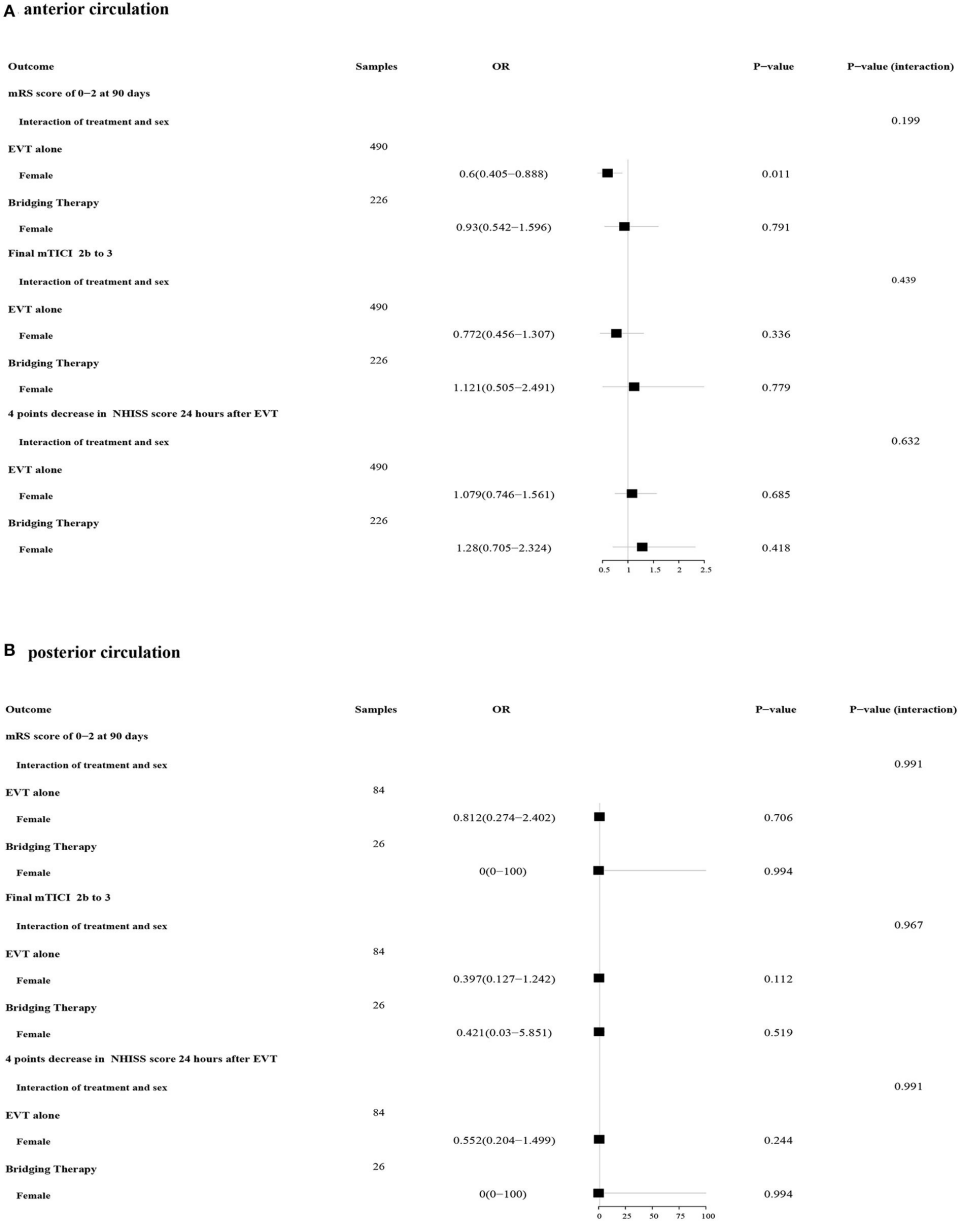
Forest plots of the association between sex and treatments in primary and secondary outcomes. **(A)** Forest plots of patients with anterior circulation ischemic stroke; **(B)** Forest plots of patients with posterior circulation ischemic stroke.

Patients were divided into two groups based on the location of the occlusion. For patients in the anterior circulation occlusion subgroup, the effect of sex on the treatments for the primary outcome was found to have differences (EVT alone: 0.6 OR, 95% CI 0.405–0.888; bridging therapy: OR 0.93, 95% CI 0.542–1.596), while no significant interaction between sex and these two treatments was discovered (P _interaction_ = 0.199) (Figure 3A). Neither sex differences nor interactions about treatment based on sex for all secondary outcomes were shown in the subgroup with anterior circulation occlusion. For patients in the posterior circulation occlusion subgroup, men were no longer observed to reach a better functional status compared with women when treated with EVT alone (OR 0.812, 95% CI 0.274–2.402). In addition, no significant interactions between sex and treatments were observed (Figure 3B). Differences in all safety outcomes were not significant between women and men regardless of the treatments and the occlusion location (Table 3).

## Discussion

According to our analysis, sex did not affect the prognosis of intravenous alteplase before EVT, but it was associated with functional independence in patients treated with EVT alone. No significant interaction between sex and treatments was found for all predefined results. The location of the occlusion did not affect the prognosis of women and men treated with bridging therapy.

Similar to Madsen et al. (18) our group found that women were less likely to be functionally independent at 90 days after being treated with EVT alone. However, this phenomenon disappeared when patients were treated with intravenous alteplase before EVT, indicating a shorter time between the onset of the disease to the onset of receiving diagnosis and treatment, and no interaction about treatment based on sex was found. Based on our baseline characteristics, men were more likely to receive bridging therapy, indicating that the time between the onset of the disease and the onset to receiving diagnosis and treatment is shorter in men. As we all know, the effect of treatment in AIS is time-dependent. Various studies demonstrated that reducing workflow time to shorten onset to treatment times was beneficial to patients (26–28). Hence, we concluded that time might have a greater impact on the effect of EVT alone compared to sex. However, there were no sex differences in the five time points at which patients were examined and underwent EVT after admission (Table 1). This might be due to the lack of other time points in our study, such as the time from the onset of disease to arrival at the hospital, which might affect the patient outcomes.

Several studies investigated the interaction between sex and different treatments in patients with AIS. According to Chalos et al. (20) the effect of EVT on the ordinal mRS was similar in women [adjusted common odds ratio (acOR), 2.13; 95% CI, 1.47–3.07] and men (acOR, 2.16; 95% CI, 1.59–2.96), with a *p*-value of 0.926 for interaction, indicating that sex does not influence the clinical outcome after endovascular treatment. In addition, several *post-hoc* analyses based on pooled data from RCTs suggest that sex does not alter the treatment effect of tPA on clinical outcomes (29–32). Interestingly, for patients with late-window stroke, sex was not associated with functional outcomes, while sex was found to influence the association between age and safety outcomes, with men experiencing worse outcomes with advancing age (33). The indirect effects of sex on efficacy and safety outcomes should be taken into consideration. Consistent with these studies, no significant interaction between sex and bridging therapy was found in our study.

de Ridder et al. (21) found the interaction between sex and intra-arterial treatment (IAT) in the MR CLEAN trial and demonstrated that men were more likely to benefit from IAT compared with women. This might be due to the broad inclusion criteria of MR CLEAN, which resulted in a population with a poor prognosis at baseline, especially in female patients. In addition, thirty patients treated with IAT underwent a simultaneous revascularization procedure, which may add complexity to the interpretation of results (34). An assessment from the Cochrane review with subgroup analysis in forest plots using data from randomized controlled trials only showed that significant interactions about sex based on treatment were slightly more common than expected by chance and had limited biological plausibility or clinical significance (35). Most of the interactions about sex based ontreatment were performed in RCTs, which were often subjected to extensive subgroup analyses without correction for multiple testing, increasing the probability of false positives. In addition, as subgroup analyses were not prespecified or insufficiently described in the protocols of RCTs, spurious findings might be reported (36). In contrast to those interaction studies, our results were based on a prospective real-world clinical practice, and confounders were selected after clinical judgment and statistical testing.

Fewer studies focused on occlusion in the posterior circulation. An analysis in patients with acute basilar artery occlusion observed that there were no significant sex differences for outcomes and recanalization, regardless of treatment modalities, including antithrombotic treatment alone, IVT or combined IVT-IAT, or IAT (37). In our study, similar results were demonstrated for patients treated with bridging therapy. However, this might be related to the small number of patients with posterior circulation ischemic stroke.

## Limitations

In this multicenter prospective, real-world study, we used propensity score for covariate adjustment to achieve approximate randomization of alteplase pretreatment. We also included patients with posterior circulation ischemic stroke and made an investigation among them. However, as we only used data from three comprehensive stroke centers in China, the conclusion lacks robustness and is difficult to extrapolate. A great number of patients were lost of follow-up at one center, which might lead to selection bias. In addition, the sample size of patients with posterior circulation ischemic stroke was small, and the confidence intervals were large when analyzing all predefined outcomes.

## Conclusion

In this multicenter prospective cohort study, we could not confirm that sex modifies the treatment effect of intravenous alteplase before endovascular therapy. Sex should not be taken into consideration when selecting patients for bridging therapy. Simultaneously, we advocate for women to seek timely medical treatment.

## Data availability statement

All data included in this study are available upon request by contacting the corresponding author.

## Ethics statement

The studies involving human participants were reviewed and approved by Shanghai Tenth People's Hospital of Tongji University. The patients/participants provided their written informed consent to participate in this study.

## Author contributions

MF was the main contributor to design, statistical analysis, and writing the first manuscript. CX was responsible for data collection. YS and LM revised the manuscript and checked the collected data. Plotting and editing analysis tables and graphs were allocated to XZ and JD. XL constructed the study and was in charge of overall direction and planning. All authors contributed to the article and approved the submitted version.

## Funding

This study was supported by Shanghai Municipal Key Clinical Specialty (shslczdzk06102).

## Conflict of interest

The authors declare that the research was conducted in the absence of any commercial or financial relationships that could be construed as a potential conflict of interest.

## Publisher's note

All claims expressed in this article are solely those of the authors and do not necessarily represent those of their affiliated organizations, or those of the publisher, the editors and the reviewers. Any product that may be evaluated in this article, or claim that may be made by its manufacturer, is not guaranteed or endorsed by the publisher.

## Supplementary material

Supplementary Figure S1 Power analysis for sample size calculation.

Supplementary Table S1 Baseline characteristics of patients who underwent EVT alone and bridging therapy.
