# Supplementary figures and images for: No sex difference was found in the safety and efficacy of intravenous alteplase before endovascular therapy
Source: Front Neurol. 2022 Nov 9;13:989166. doi: 10.3389/fneur.2022.989166 (PMC9681809; doi:10.3389/fneur.2022.989166)

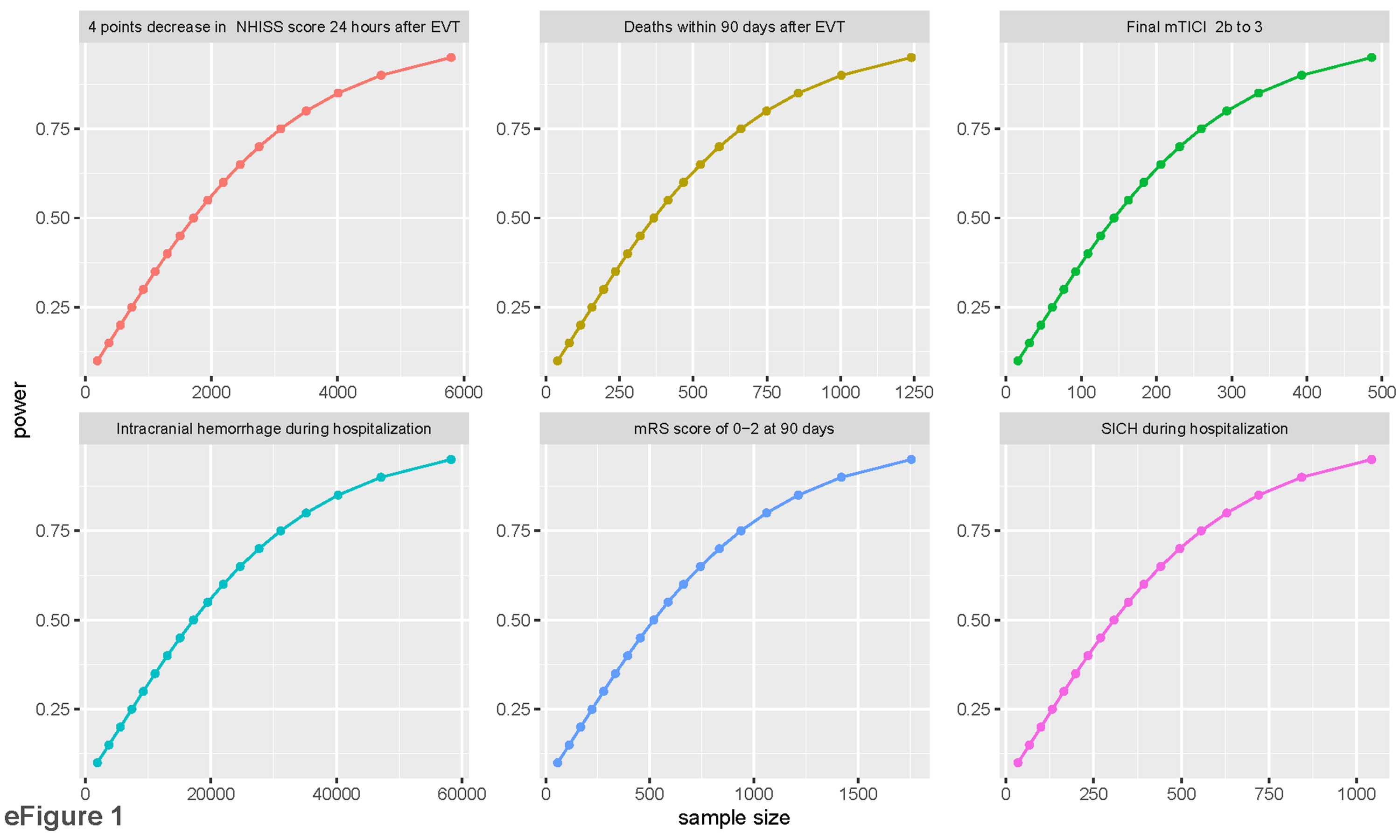

Supplement: Supplementary Figure S1 — Power analysis for sample size calculation. [file Image_1.TIF]

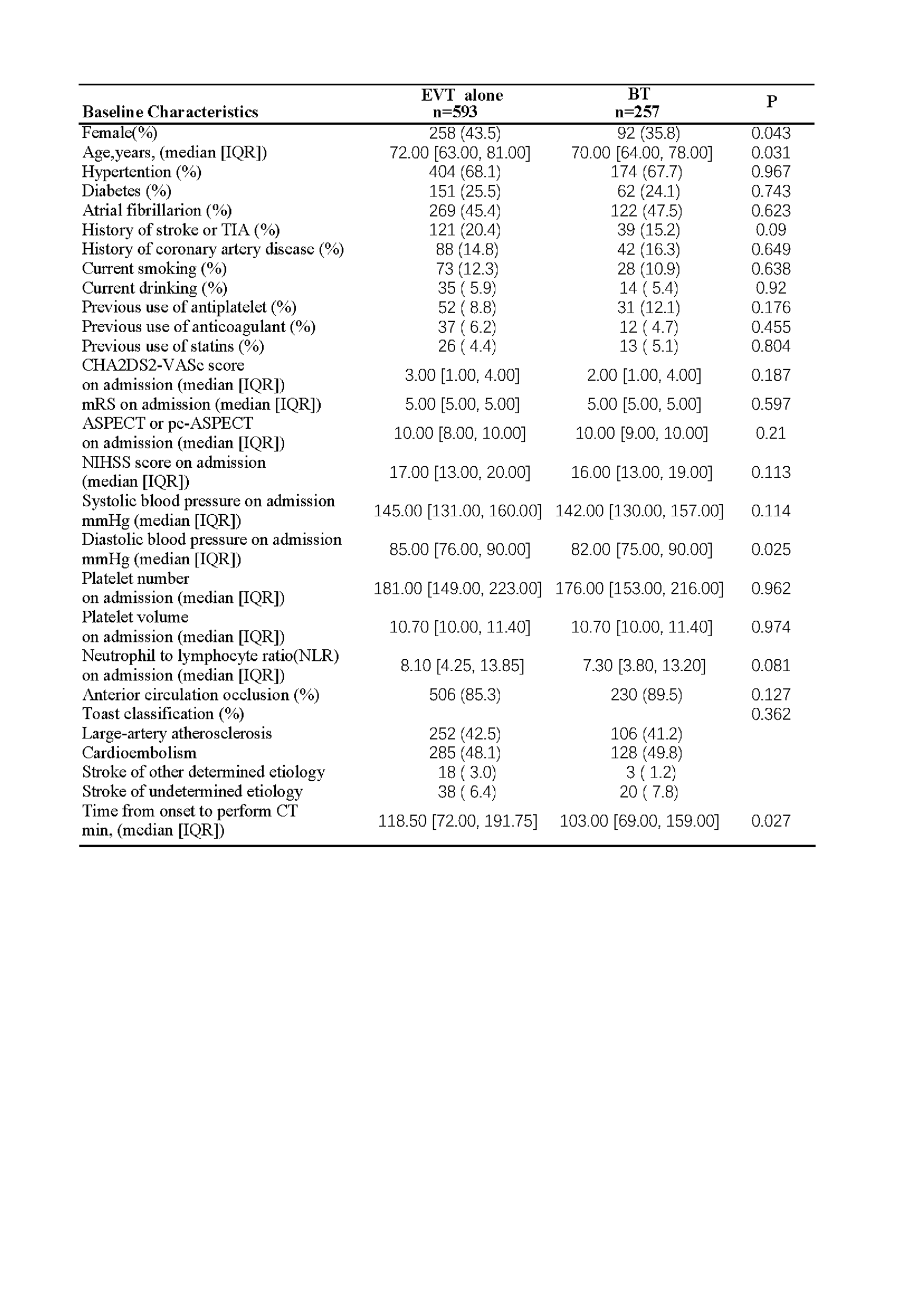

Supplement: Supplementary Table S1 — Baseline characteristics of patients who underwent EVT alone and bridging therapy. [file Image_2.TIF]
